# Immunogenicity and 1-year boostability of a three-dose intramuscular rabies pre-exposure prophylaxis schedule in adults receiving immunosuppressive monotherapy: a prospective single-centre clinical trial

**DOI:** 10.1093/jtm/taac148

**Published:** 2022-12-08

**Authors:** Hannah M Garcia Garrido, Bridget van Put, Sanne Terryn, Cornelis A de Pijper, Cornelis Stijnis, Geert R D’Haens, Phyllis I Spuls, Marleen G van de Sande, Steven van Gucht, Martin P Grobusch, Abraham Goorhuis

**Affiliations:** Amsterdam UMC, Center for Tropical Medicine and Travel Medicine, Department of Infectious Diseases, University of Amsterdam, Amsterdam Institute for Infection and Immunity, Amsterdam 1081 HZ, The Netherlands; Amsterdam UMC, Center for Tropical Medicine and Travel Medicine, Department of Infectious Diseases, University of Amsterdam, Amsterdam Institute for Infection and Immunity, Amsterdam 1081 HZ, The Netherlands; Sciensano, Brussels 1050, Belgium; Amsterdam UMC, Center for Tropical Medicine and Travel Medicine, Department of Infectious Diseases, University of Amsterdam, Amsterdam Institute for Infection and Immunity, Amsterdam 1081 HZ, The Netherlands; Amsterdam UMC, Center for Tropical Medicine and Travel Medicine, Department of Infectious Diseases, University of Amsterdam, Amsterdam Institute for Infection and Immunity, Amsterdam 1081 HZ, The Netherlands; Amsterdam UMC, Department of Gastroenterology, University of Amsterdam, Amsterdam 1081 HZ, The Netherlands; Amsterdam UMC, Department of Dermatology, University of Amsterdam, Amsterdam 1081 HZ, The Netherlands; Amsterdam UMC, Department of Rheumatology and Clinical Immunology, University of Amsterdam, Amsterdam 1081 HZ, The Netherlands; Sciensano, Brussels 1050, Belgium; Amsterdam UMC, Center for Tropical Medicine and Travel Medicine, Department of Infectious Diseases, University of Amsterdam, Amsterdam Institute for Infection and Immunity, Amsterdam 1081 HZ, The Netherlands; Amsterdam UMC, Center for Tropical Medicine and Travel Medicine, Department of Infectious Diseases, University of Amsterdam, Amsterdam Institute for Infection and Immunity, Amsterdam 1081 HZ, The Netherlands

**Keywords:** preexposure prophylaxis, boostability, schedule, rabies, vaccine, immunocompromised host

## Abstract

**Background:**

For immunocompromised patients (ICPs), administration of rabies immunoglobulins (RIG) after exposure is still recommended regardless of prior vaccination, due to a lack of data. We aimed to assess the 1-year boostability of a three-dose rabies pre-exposure prophylaxis (PrEP) schedule in individuals using immunosuppressive monotherapy.

**Methods:**

In this prospective study, individuals on immunosuppressive monotherapy with a conventional immunomodulator (cIM) or a TNF-alpha inhibitor (TNFi) for a chronic inflammatory disease received a three-dose intramuscular PrEP schedule (days 0,7,21–28) with 1 mL Rabipur®, followed by a two-dose simulated post-exposure prophylaxis (PEP) schedule (days 0,3) after 12 months. Rabies neutralizing antibodies were assessed at baseline, on day 21–28 (before the third PrEP dose), day 60, month 12 and month 12 + 7 days. The primary outcome was 1-year boostability, defined as the proportion of patients with a neutralizing antibody titre of ≥ 0.5 IU/mL at month 12 + 7 days. Secondary outcomes were geometric mean titres (GMTs) and factors associated with the primary endpoint.

**Results:**

We included 56 individuals, of whom 52 completed the study. The 1-year boostability was 90% (47/52) with a GMT of 6.16 (95% CI 3.83–9.91). All participants seroconverted at some point in the study. Early response to PrEP (at day 21–28) was significantly associated with 100% boostability (Odds Ratio 51; 95% confidence interval [5.0–6956], *P* < 0.01). The vaccination schedule was safe and well tolerated. No vaccine-related serious adverse events occurred.

**Conclusion:**

In patients using immunosuppressive monotherapy, a three-dose rabies PrEP schedule followed by a two-dose PEP schedule is immunogenic, with all patients seroconverting at some point in the study. Although boostability 7 days after PEP was not 100%, nobody would wrongly be denied RIG when only administered to those who responded early to PrEP while reducing the administration of RIG by 73%.

## Introduction

Rabies is a viral zoonosis that accounts for an estimated 59 000 human deaths and the loss of 3.7 million disability-adjusted life years annually.^1^

After a potential rabies exposure, administration of adequate post-exposure prophylaxis (PEP) can entirely prevent the development of clinical rabies and rabies-associated mortality.^2^^,^^3^ For healthy non-immunized individuals, in case of a grade III (transdermal haemorrhaging wound) or a bat-derived grade II exposure (superficial abrasion),^3^ intradermal (ID) or intramuscular (IM) PEP consists of at least three vaccinations and additional rabies immunoglobulins (RIG). The availability of RIG in rabies endemic countries is limited.^4^ Therefore, pre-exposure prophylaxis (PrEP), consisting of at least two vaccinations, is recommended for individuals travelling to endemic areas where timely administration of RIG after exposure cannot be guaranteed.^3^^,^^4^ Rabies vaccines are highly immunogenic in healthy individuals, and only two doses of PrEP induce robust immunological memory, ensuring rapid recall antibody responses 7 days after boosting with PEP, a concept known as ‘boostability’.^5^^,^^6^ Therefore, an abbreviated two-dose PEP schedule without RIG suffices in previously vaccinated healthy individuals.^3^

In contrast, data regarding immunogenicity of rabies vaccines among immunocompromised patients (ICPs) are scarce to non-existent, and mostly originate from case reports and case series.^7–15^ In HIV-infected individuals, seroconversion occurs more frequently in those with sufficient CD4-cell counts.^8^^,^^10–12^^,^^16^ Therefore, HIV-patients treated with cART and with CD4-counts above 200 cells/mm^3^ are regarded as immunocompetent.^4^ Among individuals receiving immunosuppressive therapy, seroconversion occurred more frequently in individuals receiving monotherapy compared with combined immunsupressive therapy.^7^^,^^9^^,^^13^^,^^15^ No studies exist on the boostability with PEP among ICPs with pre-exposure vaccination.

Due to this knowledge gap, the Word Health Organization recommends that ICPs who suffered a grade II or III rabies exposure must receive a full PEP schedule including RIG, regardless whether or not PrEP has been previously administered.^3^ As a consequence, rabies vaccine coverage among ICPs is low, putting this group especially at risk.^1^^,^^17^^,^^18^ This is problematic because ICPs who are treated with immunosuppressive therapy generally feel healthy and frequently embark on international travel, including to rabies-endemic areas. The majority of these ICPs comprises those with auto-immune diseases, as the ongoing development of therapies has lowered disease burden and has increased quality of life.^17^^,^^18^

The high potency of rabies vaccine in healthy individuals, combined with previously documented promising immunogenicity of rabies PEP schedules and other vaccines in individuals using TNF-alpha inhibitors, methotrexate and thioguanines, has motivated us to conduct the present study.^5^^,^^14^^,^^19^^,^^20^ We hypothesized that among patients using immunosuppressive monotherapy, a three-dose intramuscular (IM) rabies PrEP schedule is sufficiently immunogenic, with quick recall responses (within 7 days) after a two-dose IM PEP schedule 12 months later (boostability). Within this scenario, the need to administer RIG as an essential part of PEP could be re-considered, reducing the costs and complexity of PEP for this specific group and potentially increasing the uptake of rabies PrEP.

## Methods

### Study design

We conducted a single-centre prospective clinical trial at the Center for Tropical and Travel Medicine between August 2020 and March 2022. The Center of Tropical Medicine and Travel Medicine is an outpatient clinic of The Amsterdam UMC, an academic hospital in Amsterdam, the Netherlands, providing pre- and post-travel healthcare for both healthy and immunocompromised individuals. This prospective clinical study was approved by the medical ethics committee of the Amsterdam UMC and registered in the Dutch trial registry (number NL9087). All participants provided written informed consent.

### Study population

We recruited participants from rheumatology, gastroenterology and dermatology outpatient clinics. Adults (18–70 years old) with a chronic auto-immune disease receiving monotherapy with either a TNF-alpha inhibitor (TNFi group), or conventional immunomodulator such as methotrexate, azathioprine and thioguanins (cIM group), and with intended travel to a rabies endemic country within the next 5 years, were asked to participate. The following exclusion criteria were applied: prior rabies vaccination, primary or other acquired immune disorder, active malignancy, allergy to any of the components of the vaccine, haemophilic disorder precluding intramuscular injection, pregnancy and/or not being able or willing to consent. We considered *n* = 50 as appropriate to quantify boostability; this would allow us to estimate the seroconversion proportion with a precision between 7 (100% seroconversion) and 29% (at 50% seroconversion) for the Clopper Pearson 95% confidence intervals (CIs), respectively. As we expected a 10% loss to follow-up, we aimed to include 55 participants.

### Study procedures

**Figure 1:**
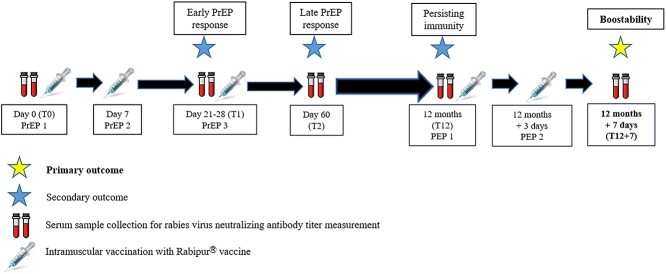
Study design. PrEP, pre-exposure prophylaxis; PEP, post-exposure prophylaxis

All participants received an IM PrEP schedule, consisting of three doses of purified chick-embryo cell culture rabies vaccine (Rabipur®, 1.0 mL, batch number ARBA581B), on days 0, 7 and 21–28, according to WHO guidelines for immunocompromised individuals.^1^^,^^3^ 1 year after enrolment, all participants received a simulated (there was no rabies exposure) two-dose IM PEP-schedule (day 0,3), to assess boostability. Vaccinations were administered in the deltoid muscle. Blood samples were taken at baseline (T0); on day 21–28, just before the third PrEP dose (T1); on day 60^±^7 days (T2); on day 365^±^20 days, just before the first PEP dose (T12); and on day 7 after the first PEP dose (T12 + 7). Serum was frozen at −80°C until further analysis (Figure 1). Samples were tested batch-wise for rabies virus neutralizing antibodies (RVNA), using the rapid focus fluorescent inhibition test (RFFIT). An adequate rabies antibody response (seroconversion) was defined as an RVNA titre of *>* 0.5 IU/mL, according to WHO recommendations.^1^ All laboratory analyses were performed in The Belgian National Rabies Reference Laboratory at Sciensano Belgian Institute of Public Health, in Brussels, Belgium.

### Safety

Adverse events (AEs) were recorded using a self-administered online questionnaire until 7 days after each vaccination. In addition participants were asked about AEs during each visit. Serious AEs were documented during the entire study period by questionnaire and chart review.

### Outcomes and statistical analysis

The primary endpoint of the study was 1-year boostability, defined as the proportion of participants with quick recall responses (RVNA *>* 0.5 IU/mL) after boosting with PEP (T12 + 7). Secondary outcomes were seroconversion rates (SCRs), geometric mean titres (GMTs) at all time points, and factors associated with seroconversion and GMT magnitude at T12 + 7. Values below the lower detection level of the RFFIT (<0.17 IU/mL) were included as a zero value in the analysis. We analysed whether there were associations between the primary outcome (boostability at T12 + 7) and sex, age, medication group, inflammatory bowel disease as underlying disease and seroconversion on T1 (early PrEP response) and T2 (late PrEP response) by estimating odds ratios with logistic regression using Firth’s bias reduction method (R version 4.1.3, package logistf). In addition, we performed univariable and multivariable linear regression analyses on log-transformed titres to examine associations between the titre magnitude at T12 + 7 and sex, age and medication group (TNFi/cIM), underlying disease (Crohn’s disease, inflammatory bowel disease, rheumatoid arthritis) and early PrEP response (T1). We used stepwise backwards selection (*P* < 0.05) to obtain the final model. We used SPSS (Chicago, Illinois, USA) version 26.0 and Graphpad Prism version 9 (California, USA) and R version 4.1.3 for all analysis. Missing data were excluded from analysis.

### Role of the funding source

This study was funded by a Research Grant from The International Society of Travel Medicine (ISTM). The funder had no role in study design; the collection, analysis and interpretation of data; in the writing of the report; nor in the decision to submit the paper for publication.

## Results

### Study population

During the study period, 56 participants were included and had provided samples eligible for analysis. Four participants withdrew prematurely, and 52 participants completed the study protocol (Supplementary Figure S1, Supplementary Materials). Baseline characteristics of patients using a TNF-alpha inhibitor or a cIM are described in Table 1. Ten participants switched treatment during the study (Supplementary Table S1, Supplementary Materials), with all switches occurring after PrEP. Two participants completely stopped using immunosuppressive drugs, one in the TNFi group and one in the cIM group. One participant appeared to have been using combination therapy with methotrexate and TNFi at baseline. However, methotrexate use only became evident after study completion. As the participant stopped using methotrexate during the study, this participant was analysed in the TNFi arm.

**Table 1:** Baseline characteristics

Characteristic	Total (*n* = 56)	cIM^a^ (*N* = 27)	TNFi^b^ (*N* = 29)
Age mean (SD^c^)	44 (15)	46 (15)	42 (14)
Age < 50 *n*/*N*(%)	34/56 (61)	16 (59)	18 (62)
Age 50–59 *n*/*N*(%)	11/56 (20)	5 (19)	6 (21)
Age 60–69 *n*/*N*(%)	11/56 (20)	6 (22)	5 (17)
Males *n*/*N* (%)	32/56 (57)	13 (48)	19 (66)
BMI mean (SD)	25.6 (4.5)	25.7 (4.1)	25.5 (4.9)
cIM *n*/*N* (%)	27/56 (48)	27/27 (100)	0/29 (0)
Methotrexate *n*/*N* (%)/median (IQR^d^) weekly dose in mg		14/27 (52)/16 (5.8)	1/29(2.3)
Azathioprine *n*/*N* (%)/mean (SD) daily dose in mg		7/27 (26)/114 (61)	NA^e^
Thioguanin *n*/*N* (%)/mean (SD) daily dose in mg		1/27 (3.7)/10(0)	NA
6-mercaptopurine *n*/*N* (%)/median (IQR) daily dose in mg		5/27 (19)/50 (31)	NA
TNFi n(%)	29/56 (52)	0/27 (0)	29/29 (100)
Infliximab *n*/*N* (%)/mean (SD) intravenous dose per 4–8 weeks in mg/kg		NA	12/29 (41)/5.5 (1.4)
Adalimumab *n*/*N* (%)/mean (SD) subcutaneous dose per 2 weeks in mg		NA	14/29 (48)/40 (0)
Etanercept *n*/*N* (%)/mean (SD) subcutaneous dose per week in mg		NA	1 (1.8)/50 (0)
Underlying diagnosis			
Crohn’s disease *n*/*N* (%)	27/56 (48)	9/27 (33)	18/29 (62)
Ulcerative colitis *n*/*N* (%)	8/56 (14)	5/27 (19)	3/29 (10)
Rheumatoid arthritis *n*/*N* (%)	4/56 (7)	4/27 (15)	0/29 (0)
Psoriasis/psoriatic arthritis *n*/*N* (%)	11/56 (20)	4/27 (15)	7/29 (24)
Other^f^ *n*/*N* (%)	6/56 (11)	5/27 (19)	1/29 (3.4)
Current smoker *n*/*N* (%)	6/56 (11)	4/27 (15)	2/29 (6.8)
Charlsson comorbidity index median (IQR)	1 (1.0)	1.0 (1.0)	1.5 (1.5)

^a^Conventional immunomodulators, ^b^TNF-inhibitors, ^c^SD, standard deviation, ^d^IQR, interquartile range,^e^NA, not applicable, ^f^Other diseases: Sarcoidosis (*n* = 2), Sjögren’s disease (*n* = 1), Axial spondyloarthritis (*n* = 1), Severe atopic eczema (*n* = 1), Alopecia areata (*n* = 1)

### Boostability, SCRs and GMTs

**Figure 2:**
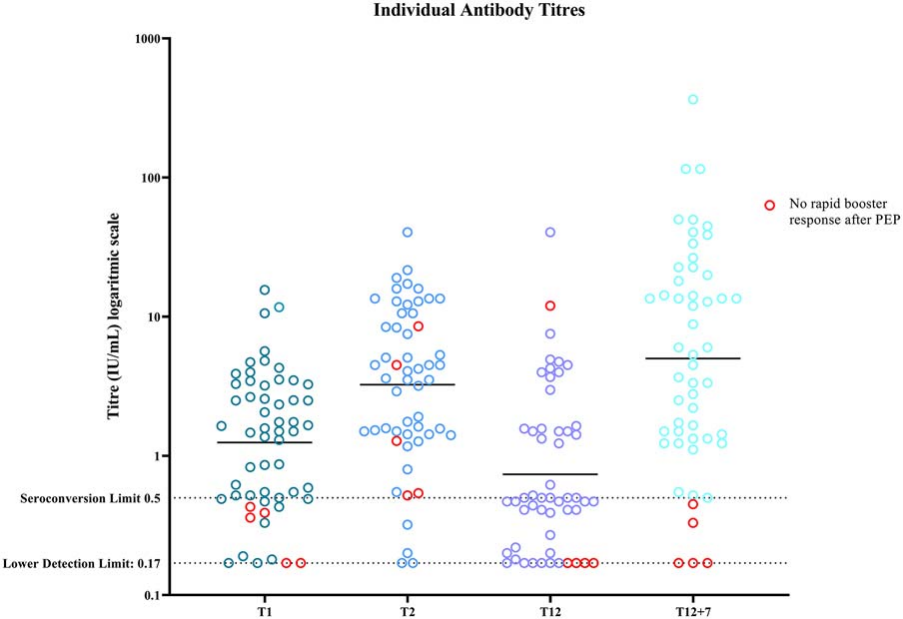
Individual antibody titres in International Units/mL. All baseline titres were below detection limit and are not shown in this figure. The black horizontal lines represent the GMTs. Values below the lower detection limit of RFFIT of < 0.17 IU/mL have been plotted as 0.17 IU/mL. PEP, post-exposure prophylaxis; T1 = days 21–28 (before the third PrEP dose); T2 = 2 months after start PrEP; T12 = 12 months after start PrEP, before PEP; T12 + 7 = 7 days after start PEP

At T12 + 7 (boostability endpoint), 90% (47/52) participants had achieved a rapid recall response (RVNA *>* 0.5 IU/mL), with a GMT of 6.16 (95%CI 3.83–9.91) (Figure 2). SCRs after PrEP at T1, T2 and T12 were 73 (41/56), 93 (50/54) and 53% (28/53), respectively (Table 2). One of the non-responders on T12 + 7 displayed a high titre of 11.98 IU/mL on T12 (before PEP). However, after re-analysing the sample with RFFIT, the titre remained below detection limit. The only factor significantly associated with the primary outcome boostability at T12 + 7 was early PrEP response at T1 (OR 51; 95% CI [5.0–6956]). This association remained significant when adjusting for all the other predefined variables; however, the fit of the model did not improve when adding covariates to the model (Table 3). Clinical characteristics of the five non-responders at the primary endpoint vary (Supplementary materials, Supplementary Table S2). There was no significant association between boostability at T12 + 7 and age, sex, medication regimen, late PrEP response or underlying diagnosis (Table 3).

**Table 2:** SCR and GMT over time

	**Time point (*T*)**	**Seroconversion *n*/*N* (%) [95%CI]**	**GMT** ^**a**^ **(95% CI**^**b**^**)**	**Range (IU/mL)**
PrEP^c^	0	0/56 (0) [0–6]	0	<0.17
	1	41/56 (73) [60–84]	1.46 (1.09–1.95)	<0.17–15.6
	2	50/54 (93) [82–98]	3.69 (2.67–5.09)	<0.17–40.5
PEP^d^	12	28/53 (53) [38–66]	1.10 (0.74–1.65)	<0.17–40.5
	12 + 7	47/52 (90) [75–95]	**6.16 (3.83–9.91)**	**<0.17–365**

^a^GMT, geometric mean titre

^b^CI (confidence interval) values below the lower detection level RFFIT (<0.17 IU/mL) were included as a zero-value in further analysis.

^c^PrEP, pre-exposure prophylaxis

^d^PEP, post-exposure prophylaxis, Bold value shows primary endpoint.

**Table 3:** Factors associated with boostability at T12 + 7 (primary outcome)

	Univariable firth logistic regression			Multivariable firth logistic regression		
**Variable**	**OR** ^**a**^	**95% CI** ^**b**^	***P*-value**	**OR**	**95% CI**	***P*-value**
**Age binary (> 50)**	0.41	0.06–2.32	0.3070	0.29	0.00–4.29	0.3854
**Male sex**	1.88	0.33–12.2	0.4691	2.03	0.22–26.7	0.5216
**TNF-alpha inhibitor**	0.70	0.11–4.19	0.7354	1.85	0.06–229	0.7109
**Inflammatory bowel disease**	1.24	0.19–7.06	0.8046	0.12	0.00–6.09	0.3182
**Early responder PrEP** ^**c**^ **T1**	**51.11**	**5.04–6956**	**0.0002**	**83.00**	**4.79–63 538**	**0.0003**
**Late responder PrEP T2**	0.88	0.01–0.93	0.9330	0.18	0.00–5.23	0.3240

^a^Odds ratio

^b^Confidence interval

^c^Pre-exposure prophylaxis, Bold value shows primary endpoint.

All of the five participants without booster response on T12 + 7 were early PrEP non-responders (Figure 2). Overall, 39/47 (83%) of boostable participants on T12 + 7 were also early PrEP responders (*P* = < 0.01), but all non-boostable participants on T12 + 7 days (*n* = 5) did achieve seroconversion to PrEP later, on T2 (late PrEP response). Two individuals did not develop seroconversion at all after PrEP but responded adequately to PEP. After boosting with two doses of PEP, a significant rise in GMTs occurred in both the TNFi and cIM group (*P* = < 0.01, data not shown).

### Factors of influence on the magnitude of antibody titres after PEP (12 months +7 days)

In univariable and multivariable linear regression analyses, increasing age, treatment with TNFi, underlying diagnosis of rheumatoid arthritis and male sex were significantly associated with lower post-PEP titres (Supplementary Table S3).

### Safety

In total, 274 vaccine doses were administered and 35 (13%) AEs were reported. Most events were reported during the PrEP schedule, with 45% of participants having reported an AE after T1. Only 9.6% of participants reported an AE after PEP. AEs were mostly transient and self-limited. Two SAEs were reported comprising two hospitalizations, due to stoma dysfunction and asthma relapse, none of which were related to the study procedures.

One participant withdrew prematurely from the study due to severe dizziness after the second PrEP-dose. The most frequently reported AEs were mild or moderate (90%) and consisted of local reactions at the injection site (20%) and musculoskeletal symptoms (14%) (Supplementary Figures S2 and Supplementary Table S4, Supplementary Materials). Relapse of underlying disease occurred in 7/56 participants (13%) with a range of 3 days–11 months after the last vaccination and comprised 20% of all AEs. In none of these cases, a relation between vaccination and relapse was suspected by the treating physician.

## Discussion

In the present study, we investigated the 1-year boostability of a three-dose rabies PrEP schedule in patients using immunosuppressive monotherapy. Although the investigated vaccination schedule was immunogenic in patients using immunosuppressive monotherapy, with all participants seroconverting at some point in the study, not all participants were able to mount a quick recall response after boosting (90%). This implies either a delayed humoral response, a dose effect or a combination of both. A delayed antibody response has previously been documented in paediatric transplant patients and in a large Australian cohort of travellers.^7^^,^^21^ Interestingly, we found that the absence of an early response to PrEP also seems to predict a delayed response to PEP. Unfortunately, we were not able to document a potentially delayed response to PEP, as our primary outcome was boostability within 7 days after PEP, and we did not measure responses at a later time point. However, from a practical standpoint, only rapid boostability to PEP is of clinical importance, as RIG should not be given after day 7 following the first rabies vaccine dose and in previously vaccinated persons it can only be omitted if a rapid immunologic response occurs after PEP. As we observed such a rapid recall immunologic response in all ICPs (*n* = 41) who seroconverted early (T1) after PrEP, we propose that RIG should not be recommended after potential rabies exposure in the first year after PrEP for early responders (positive antibodies on the day of the third PrEP dose) among patients using immunosuppressive monotherapy.

We report a higher SCR compared with a recent observational study, which found an SCR of 78% in a retrospective study of in total 28 individuals, after a four- or five-dose PEP-schedule in non-immunized ICPs using immunosuppressive monotherapy.^14^ This indicates that PrEP not only increases the response to PEP in patients using immunosuppressive drugs, but also provides the rapid booster effect necessary to consider not administrating RIG after a potential rabies exposure, in the majority of participants.

As GMTs after boosting were much lower in our cohort compared to GMT reported in healthy individuals (6.16 IU/mL vs 17.64 IU/mL (ID)—59.87 IU/mL (IM)),^5^ life-long boostability as has been demonstrated for healthy individuals cannot be assumed and has to be investigated in future cohorts. Increasing age, treatment with TNFi, rheumatic arthritis as underlying disease and male sex were significant predictors for lower post-booster RVNA. Prior studies reported a similar negative effect of increasing age on GMT magnitude, congruent to our assessment, but a positive effect of TNF-alpha inhibitor use.^14^^,^^21^^,^^22^ This could be due to the smaller sample size of this study.

### Strengths and limitations

The present study is, to the best of our knowledge, the first prospective study to assess immunogenicity and boostability of rabies PrEP followed by simulated PEP in patients with immunosuppressive monotherapy. There are several limitations. First, the sample size was small, and it may be necessary to confirm our findings in a larger cohort. On the other hand, the WHO decided to regard HIV patients on antiretroviral therapy and CD4 count > 200 cells/mm^3^ as immunocompetent based on even smaller studies.^3^ Second, we did not investigate more extensive PEP schedules, which may have led to 100% seroconversion, nor did we assess RVNAs beyond month 12 + 7 days, as a delayed response to PEP may likely have occurred. Last, we suspect that in one of the non-responders on T12 + 7 days, the RFFIT may have been false negative, as the titre of this patient on T2 and T12 was high. Retesting, however, yielded the same (negative) result. Possibly, a sample mix-up (between T12 and T12 + 7) occurred in the laboratory. We may therefore have overestimated the proportion of non-responders at T12 + 7. However, considering this participant as PEP responder did not strongly influence the SCR and other outcomes in sensitivity analyses.

### Recommendations for clinical practice and future studies

Based on the data presented here, the additional value of RIG as part of PEP should be reconsidered for individuals on immunosuppressive therapy who respond early to PrEP within the first year after vaccination. Future studies are necessary to determine whether long-term protection can be achieved.

In the meantime, the important question remains what to offer ICPs who received rabies PrEP longer than 1 year ago. In this scenario, a practical approach for travel clinics could be to administer a rabies booster dose prior to departure, and measure the antibody response 7 days later. A satisfactory response could then be used as an indication that administration of RIG after a potential exposure to rabies is not necessary.

If a policy to leave out RIG for ICPs who respond adequately (<7 days) to PEP would be applied to our study population of ICPs on immunosuppressive monotherapy, no participant would wrongly be denied RIG while reducing the unnecessary administration of RIG by 73%.

In addition, as all non-responders after PrEP were able to achieve seroconversion at some point in the study, higher dosed PrEP/PEP schedules may have the potential to induce 100% seroconversion. For example, in previously conducted studies regarding HIV infected individuals, an SCR of 100% was documented after administration of a double dosed ID regimen, compared with an SCR of 70% after a normal-dosed ID regimen, independently of CD4 count.^10^^,^^11^ Likewise, an additional IM dose resulted in seroconversion in all patients using immunosuppressive therapy who initially responded insufficiently after regular PEP.^14^ Therefore, pilot studies investigating double dose ID PrEP followed by double dose ID PEP schedule in patients using immunosuppressive drugs are recommended.

In conclusion, although a three-dose rabies PrEP schedule followed by a two-dose simulated PEP schedule was immunogenic in individuals receiving immunosuppressive monotherapy, the goal of 100% boostability 7 days after PEP was not achieved. The ability to predict boostability after PEP by the measurement of early PrEP-response is promising and could serve as a discriminating tool for RIG administration in clinical practice. Future studies must elucidate the duration of boostability in patients using immunosuppressive therapy.

## Author contributions

H.M.G., C.A.d.P., A.G., M.P.G. and C.S. conceived the study. C.A.d.P. performed the literature search before the study. H.M.G. and C.A.d.P. wrote the study protocol. H.M.G. recruited patients together with G.R.D., M.G.v.d.S. and P.I.S. H.M.G. and B.v.P. performed data collection under the supervision of A.G. B.v.P. made the figures. S.T. and S.V.G. were responsible for laboratory analyses. B.v.P. performed statistical analyses together with H.M.G. B.v.P., H.M.G. and A.G. wrote the first version of the manuscript. All authors contributed to the data interpretation and the writing of the final manuscript. All authors had full access to all the data in the study and accept responsibility to submit for publication.

## Data Availability statement

The datasets used during the current study are available from the corresponding author on reasonable request.

## Supplementary Material

Supplementary_materials_CADP_taac148
