# Supplementary material for: Immunogenicity and 1-year boostability of a three-dose intramuscular rabies pre-exposure prophylaxis schedule in adults receiving immunosuppressive monotherapy: a prospective single-centre clinical trial
Source: J Travel Med. 2022 Dec 8;30(2):taac148. doi: 10.1093/jtm/taac148 (PMC10075057; doi:10.1093/jtm/taac148)
Supplement: Supplementary_materials_CADP_taac148 [file supplementary_materials_cadp_taac148.docx]

Supplementary Materials

**Figure S1: Study Flowchart**


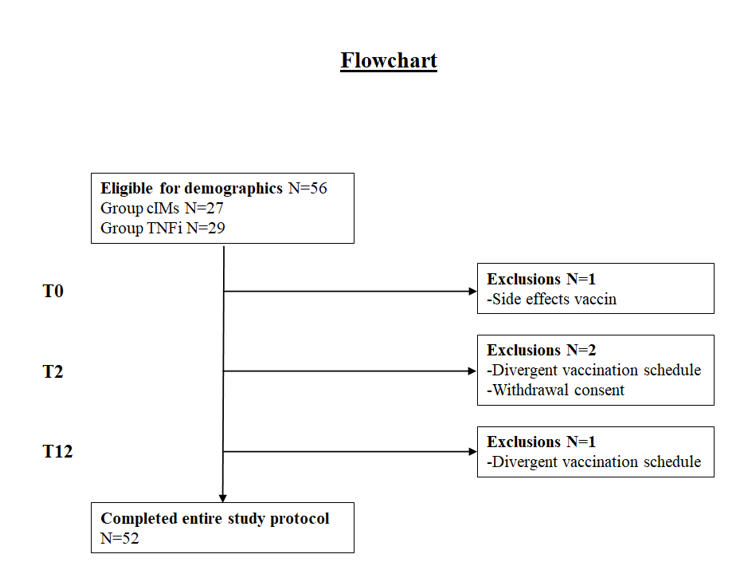


cIMs: conventional immunomodulators

TNFi= TNF-alpha inhibitor

***Figure S2 Adverse events***

 Other: transaminitis (n=2, not attributed to the study protocol by treating physician), agitated sensation (n=1), acute vertigo (n=1), COVID-19 (n=1).

**Table S1. Medication Switches**

| Participant | Old regime | New regime | Time after last vaccination | Time before next vaccination | Vaccines received |
| --- | --- | --- | --- | --- | --- |
| 1 | Methotrexate + Adalimumab | Adalimumab | 5 months | 5 months | 3 |
| 2 | Adalimumab | Guselkumab | 9 months | 1.5 months | 3 |
| 3 | Adalimumab | Secukinumab | 2 months | 9 months | 3 |
| 4 | Infliximab | None | 1.5 months | 9 months | 3 |
| 5 | Adalimumab | Ustekinumab | 4 months | 6.5 months | 3 |
| 6 | Adalimumab | Risankizumab | 4 months | 7 months | 3 |
| 7 | Methotrexate | Upadacitinib | **10 months** | **1.5 months** | 3 |
| 8 | Methotrexate | Adalimumab | 8 months | 3 months | 3 |
| 9 | Azathioprine | Infliximab + Azathioprine | 2.5 months | 8.5 months | 3 |
| 10 | Azathioprine | None | **4 days** | **10,5 months** | 3 |
| Borders of range have been highlighted. | | | | | |

**Table S2. Characteristics of patients without rapid recall response to PEP (boostability) at T12+7.**

|  |  |  |  |  |  |  |  |
| --- | --- | --- | --- | --- | --- | --- | --- |
| Gender/age | | Country of origin | Underlying disease | Medication regime | Titre (IU/mL) | Medication switch | Timing 3^rd^ PrEP^1^ dose |
| Female/65 | | The Netherlands | Crohn’s disease | Adalimumab | <0.17 | No | Day 21 |
| Male/41 | | The Netherlands | Crohn’s disease | Infliximab | 0.45 | No | Day 21 |
| Female/53 | | The Netherlands | Rheumatoid arthritis | Methotrexate | 0.33 | No | Day 28 |
| Male/55 | | The Netherlands | Psoriatric arthritis | Methotrexate | <0.17 | No | Day 28 |
| Female/32 | | The Netherlands | Crohn’s disease | Infliximab | <0.17 | No | Day 28 |

^1^ PrEP = Pre-exposure prophylaxis

**Table S3: Factors associated with post-booster titer magnitude at 12 months and 7 days in univariable and multivariable linear regression analysis.**

|  | Univariable (B+95% CI) | p-value | Multivariable  (B+95% CI) | p-value | |
| --- | --- | --- | --- | --- | --- |
| Age (years) | -1.04 (-1.07- -0.99) | **0.021** | -1.04 (-1.07- -1.01) | **0.009** | |
| Sex^1^ | 3.01 (1.10-6.97) | **0.032** | 2.76 (1.24-6.10) | **0.014** | |
| Medication group^2^ | 2.83 (1.14-7.04) | **0.026** | 3.96 (1.75-8.96) | **0.001** | |
| Rheumatoid Arthritis^3^ | -4.41 (-24.14-1.24) | 0.086 | -6.71 (-31.25- -1.44) | **0.017** | |
| Crohn’s Disease^3^ | - 1.06 (-2.79-2.46) | 0.898 | *NS^5^* | | |
| Early PrEP^4^ response (T1)^3^ | 2.05 (-1.57-6.64) | 0.224 |  |  |  |
| Inflammatory bowel disease | -1.60 (-4.30-1.68) | 0.342 |  |  |  |
| Statistically significant values have been highlighted. ^1^Reference = male, ^2^Reference=TNFi, ^3^ Reference = No Rheumatoid arthritis/ No Crohn’s Disease/ No inflammatory bowel Disease/ No early PrEP response at T1, ^4^ PrEP = Pre-exposure prophylaxis ^5^ NS = Non significant and excluded by stepwise backwards selection model. Statistically significant outcomes have been highlighted in bold. | | | | |  |

**Table S4: Adverse events in prevalence and intensity**

| Related to study protocol | % (total number of events) |
| --- | --- |
| Not related | 3% (1) |
| Unlikely | **40% (14)** |
| Possible | 11% (4) |
| Likely | **37% (13)** |
| Certain | 9% (3) |

| Time point AE in months after first PrEP dose (T) | % (total number participants) |
| --- | --- |
| T1 (PrEP) | **44.6% (25/56)** |
| T3 | 1.9% (1/54) |
| T5 | 1.9% (1/54) |
| T8 | 1.9% (1/54) |
| T12 (PEP) | **9.6% (5/52)** |
| T19 (12+7 days) | 1.9% (1/52) |
| T13 | 1.9% (1/52) |
| Most frequently occurring time points have been highlighted | |

PrEP = Pre-exposure prophylaxis

PEP= Post-exposure prophylaxis
